# The Effects of Antioxidant Cocktail on Ophthalmological Changes Induced by a 60-Day Head-Down Bed Rest in a Randomized Trial

**DOI:** 10.3390/life14121598

**Published:** 2024-12-04

**Authors:** Marc Kermorgant, Fanny Varenne, Anne Pavy-Le Traon, Thomas Geeraerts, Lisa Barioulet, Pierre Fournié, Rebecca Billette de Villemeur, Marie-Pierre Bareille, Arnaud Beck, Adrianos Golemis, Inês Antunes, Guillemette Gauquelin-Koch, Vincent Soler, Jean-Claude Quintyn

**Affiliations:** 1 UMR INSERM U1297, Institute of Cardiovascular and Metabolic Diseases (I2MC), 31432 Toulouse, France; marc.kermorgant@gmail.com; 2 Department of Ophthalmology, University Hospital of Toulouse, 31059 Toulouse, France; 3 Department of Neurology, University Hospital of Toulouse, 31059 Toulouse, France; 4 Department of Anaesthesiology and Critical Care, University Hospital of Toulouse, 31059 Toulouse, France; 5 Institute for Space Medicine and Physiology (MEDES), 31405 Toulouse, France; 6 Telespazio Belgium S.R.L. for the European Space Agency, NL-2200 AG Noordwijk, The Netherlands; 7 Centre National d’Etudes Spatiales (CNES), 75001 Paris, France; 8 Department of Ophthalmology, Unicaen, University Hospital of Caen, 14033 Caen, France

**Keywords:** optical coherence tomography, optic nerve sheath diameter, intraocular pressure, head-down bed rest, countermeasure, microgravity

## Abstract

Neuro-ophthalmological changes have been reported after prolonged exposure to microgravity; however, the pathophysiology remains unclear. Furthermore, several countermeasures have been suggested to counteract the side effects of microgravity. The objectives of the present study were twofold: (1) to assess the neuro-ophthalmological impact of 60 days of head-down bed rest (HDBR) and (2) to determine the potential effects of an antioxidant cocktail. In this case, 20 healthy male subjects completed a 60-day HDBR and were randomly allocated into two groups: a control condition without an antioxidant cocktail (CON) and a condition with an antioxidant cocktail (NUT). The retinal nerve fibre layer thickness (RNFLT) and central retinal thickness (CRT) were assessed with spectral domain optical coherence tomography. The optic nerve sheath diameter (ONSD) was measured by ocular ultrasonography and used to assess indirect changes in the intracranial pressure (ICP). The intraocular pressure (IOP) was assessed by Goldmann applanation tonometry. The CRT tended to be reduced after HDBR. The ONSD was increased at the end and after HDBR. The IOP tended to decrease after HDBR. Finally, the antioxidant cocktail had minor impacts on the ophthalmological changes induced by HDBR. It is worth noting that two participants presented peripapillary edema.

## 1. Introduction

Spaceflight-Associated Neuro-ocular Syndrome (SANS) involves a plethora of ophthalmic abnormalities seen after long-duration spaceflight, and some astronauts present unilateral and bilateral optic disc edema, globe flattening, choroidal and retinal folds, hyperactive shifts or cotton wool spots [1,2]. Although precise SANS mechanisms are not well known, this syndrome may be due to intracranial fluid accumulation following a headward fluid shift. Hence, the onset of these ophthalmological changes during spaceflight should not be ignored.

Spaceflight analogue environments such as head-down bed rest (HDBR), dry immersion and parabolic flight are under controlled environments to determine the impact of microgravity on various physiological systems [3]. HDBR is one of the most commonly used ground-based analogues to determine the impact of microgravity on the human body (body unloading, cranial fluid shift, immobilization, cardiovascular deconditioning, etc.). This model consists of placing the subject lying down on a bed tilted at 6° with a head-down orientation. As observed in spaceflight, the onset of cardiovascular deconditioning and a decreased exercise capacity occur at the end of HDBR. However, it is worth noting that, unlike spaceflight, HDBR does not remove the gravitational force acting from the chest to the back (Gx) [4,5]. Antioxidants have been proposed as an alternative countermeasure to lessen bone loss after microgravity exposure [6]. Whether alone or combined, an exercise and dietary regimen had limited protective effects on the bone and muscle structure in astronauts [7]. However, the effects of HDBR combined with antioxidant intake on ophthalmological changes remained unclear.

The aims of this study were:(1) to assess the neuro-ocular modifications induced by a 60-day HDBR and (2) to determine whether the antioxidant cocktail has an impact on these HDBR-induced ophthalmological adaptations. We hypothesized that a thoraco-cephalic fluid shift, induced by HDBR, would lead to ocular changes.

## 2. Materials and Methods

### 2.1. Subjects

The clinical trial (ID-RCB 2016-A00401-50; Clinical Trial Identifier: NCT03594799) was carried out in accordance with the principles laid down by the Declaration of Helsinki after approval by both the CPP Sud Ouest et Outre Mer I Ethic Committee and the French Health Authority, ANSM. The authors confirm that all ongoing and related trials for this intervention are registered. The participant recruitment was split into two campaigns. Twenty healthy men (at selection: 34 ± 8 years; 176 ± 5 cm; 74 ± 7 kg) were included in the study and provided their written informed consent. The inclusion and non-inclusion criteria were identical to those presented in a previous study [8]. None of the participants presented ophthalmology defects prior to study.

### 2.2. General Protocol

**Figure 1:**
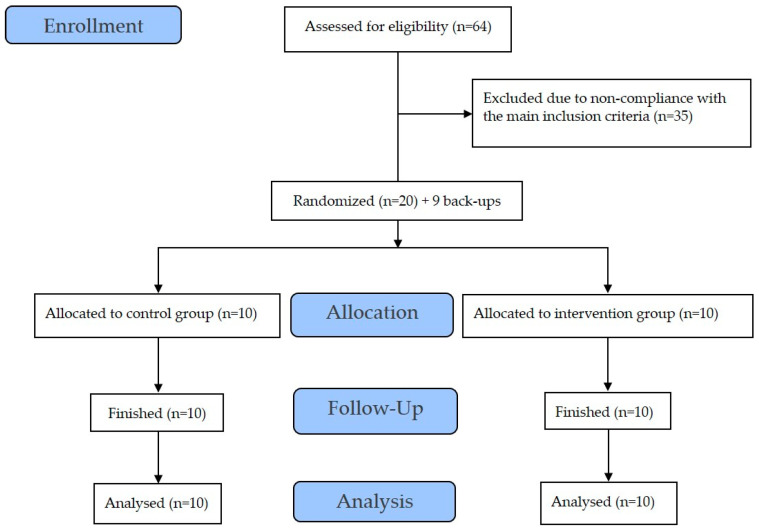
Flow chart of the study.

The study was conducted at the Institute for Space Medicine and Physiology (MEDES-IMPS) in Toulouse (France) and was separated into 2 campaigns (with 10 subjects per campaign). The volunteers were randomly allocated into 2 groups: a control condition without antioxidant cocktail (CON) and a condition with antioxidant cocktail (NUT). The study was sponsored by the European Space Agency (ESA) and the French Space Agency [Centre National d’Etudes Spatiales (CNES)]. Each volunteer participated in one study campaign for a total duration of 88 days, including 14 days for ambulatory control period (BDC-14 to BDC-1), 60 days for HDBR period (HDBR+1 to HDBR+60) and 14 days for recovery period (R 0 to R+13). A flow diagram of the study is shown in Figure 1.

### 2.3. Antioxidant Cocktail

A bioactive polyphenol compound (flavonols: 323.4 mg, phenylpropanoids: 45.6 mg, oligostilbenes: 78.0 mg, hydroxycinnamic acids: 50.4 mg, flavanols: 135.6 mg, flavanones: 108.0 mg) (XXS-2A-BR2; Spiral Company, Dijon, France) was daily administered, divided into 6 pills per day (equivalent to a daily dose of 741 mg), with 2 pills at breakfast, 2 at lunch and 2 at dinner, consisting of 168 mg of vitamin E with 80 µg of selenium (Solgar, Marne-La-Vallée, France) and 2.1 g of omega-3 fatty acids (Omacor^®^, Pierre Fabre, Toulouse, France).

### 2.4. Optical Coherence Tomography

The eyes of each participant were imaged using Spectralis OCT (Heidelberg Engineering software version 5.1.3.0, GmbH, Heidelberg, Germany). The measurements were performed at rest in seated position during BDC-1 and R+5 examinations with automatic follow-up scan placement by trained ophthalmologists. OCT was performed to quantify the RNFLT, as a reflection of a disc swelling. The quality of each measurement was determined by a quality index provided by the OCT device. Measurements not fulfilling this condition were automatically eliminated and repeated. ON head thickness was divided into four quadrants: temporal, nasal, superior and inferior. Right and left eyes were assessed. The final measure corresponds to the average of the two measures. Central retinal thickness (CRT) was evaluated with a 30° × 25° OCT macular cube, centred on the fovea, including 61 consecutive horizontal B-scans. All OCT measurements were validated by experts (Fanny Varenne, Vincent Soler and Jean-Claude Quintyn) blinded to the condition.

### 2.5. Optic Nerve Sheath Diameter

Ocular examination was performed in 10 subjects (5 in each group) at rest in supine position during BDC-5, HDBR+3, HDBR+20, HDBR+57 and R+13 by investigators with previous experience in ocular ultrasonography. Ultrasound was performed with a linear high-frequency probe (OrcheoLite, Sonoscanner, Paris, France). The subjects were asked to avoid eye movements. ONSD was measured 3 mm behind the posterior globe. Each measurement was performed for each eye. The mean ONSD value was calculated between right and left ON. All ONSD measurements were validated by an expert investigator (Thomas Geeraerts) blinded to the condition.

### 2.6. Intraocular Pressure

One drop of tetracaïne (1%), a topical local anesthetic, was administered to each eye just prior to the IOP measurement. The IOP measurements were performed with a Goldmann^®^ (Munich, Germany) applanation tonometry at rest in supine position during BDC-1 and R+5. Then, the two measurements were averaged to obtain a final measure.

### 2.7. Statistical Analysis

First, Shapiro–Wilk test was performed to test the normality of the data. OCT and IOP data were normally distributed, while ONSD (NUT group) was abnormally distributed. Data were analyzed using software GraphPrism 9 by two-way ANOVA with post hoc Šídák’s test (OCT and IOP data) to assess the effect of time spent in HDBR condition and the impact of the antioxidant cocktail. Mann–Whitney U test was applied to compare ONSD data between CON and NUT groups. Thereafter, one-way ANOVA with repeated measures with Dunnett’s multiple comparisons tests and Friedman test with Dunn’s multiple comparisons tests were used for ONSD data, respectively, in CON and NUT groups. *p* values of <0.05 were considered statistically significant.

## 3. Results

### 3.1. Optical Coherence Tomography

**Figure 2:**
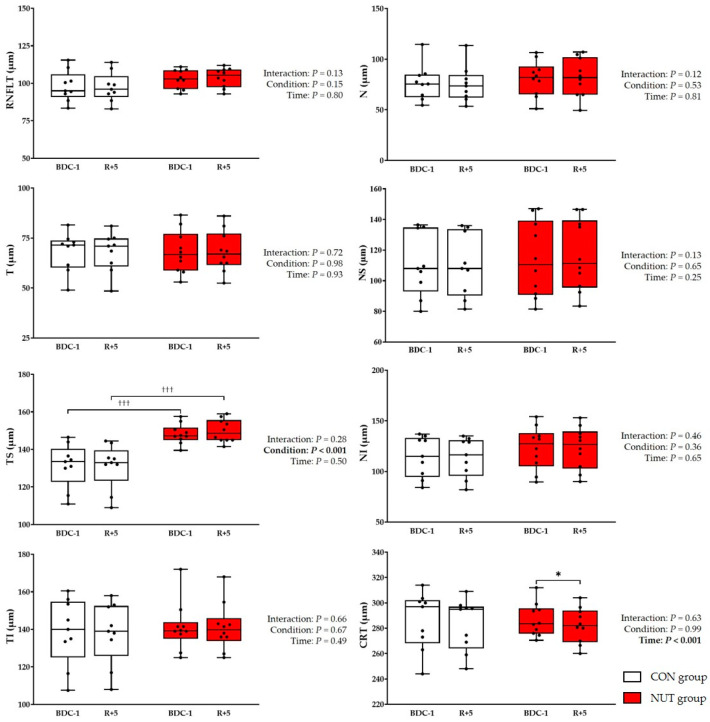
Optical coherence tomography data before (BDC-1) and after (R+5) head-down bed rest. CRT: central retinal thickness, N: nasal, NI: nasal inferior, NS: nasal superior, RNFLT: retinal nerve fibre layer thickness, T: temporal, TI: temporal inferior, TS: temporal superior. Black dots represent individual points. The box represents the interquartile range from the 25th to 75th percentile. The middle line represents the median. The whiskers extend from the minimum to the maximum value. †††: *p* < 0.001 vs. the same period in CON group, *: *p* < 0.05 vs. BDC-1 in NUT group.

In the CON group, one subject was removed from OCT analysis due to non-reliable values. We observed a thinner RNFL in the temporal superior quadrant in the CON group than in the NUT group; however, the RNFLT in all quadrants were preserved at R+5. In the CON group, the CRT tended to decrease at R+5 (*p* = 0.09); while in the NUT group, the CRT dwindled (*p* = 0.02). It is worth noting that in the CON group, eight of the nine volunteers presented a decreased CRT (Figure 2). In the NUT group, two subjects presented peripapillary edema; however, they recovered 1 and 2 months later (assessed during the medical follow-up).

### 3.2. Optic Nerve Sheath Diameter

The Mann–Whitney U test did not reveal any significant differences between the CON and NUT groups.

**Figure 3:**
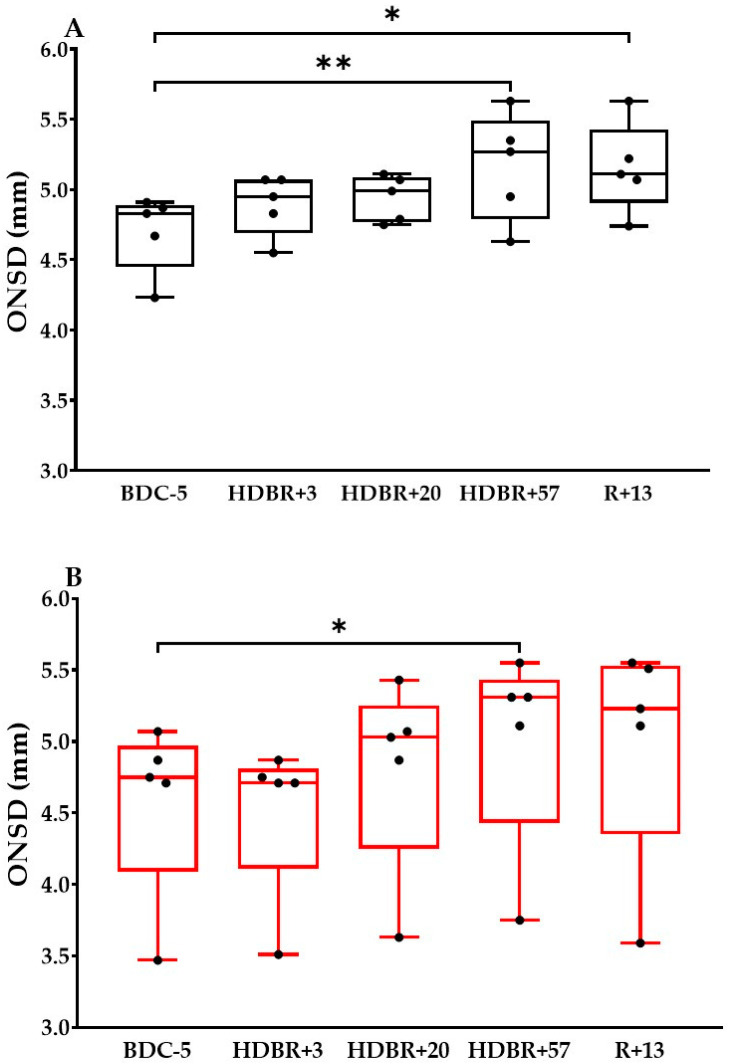
Optic nerve sheath diameter (ONSD) measurements before (BDC-5), during (HDBR+3, HDBR+20, HDBR+57) and after (R+13) head-down bed rest in control (**A**) and antioxidant cocktail (**B**) groups. Black dots represent individual points. The box represents the interquartile range from the 25th to 75th percentile. The middle line represents the median. The whiskers extend from the minimum to the maximum value. * *p* < 0.05 vs. BDC-5, ** *p* < 0.01 vs. BDC-5.

In the CON group, the ONSD did not change significantly either at HDBR+3 (4.9 ± 0.2 mm; *p* = 0.61) or at HDBR+20 (4.9 ± 0.2 mm; *p* = 0.29); however, the ONSD increased significantly by 11% during HDBR+57 (5.2 ± 0.4 mm; *p* < 0.01) and remained higher by 11% at R+13 (5.2 ± 0.3 mm; *p* = 0.02) compared to BDC-5 (4.7 ± 0.3 mm) (Figure 3A).

In the NUT group, the ONSD did not change significantly either at HDBR+3 (4.5 ± 0.6 mm; *p* > 0.99) or at HDBR+20 (4.8 ± 0.7 mm; *p* > 0.99); however, the ONSD increased significantly by 9% during HDBR+57 (5.0 ± 0.7 mm; *p* = 0.04) but was not significantly modified at R+13 (5.0 ± 0.8 mm; *p* = 0.11) compared to BDC-5 (4.6 ± 0.6 mm) (Figure 3B).

### 3.3. Intraocular Pressure

**Figure 4:**
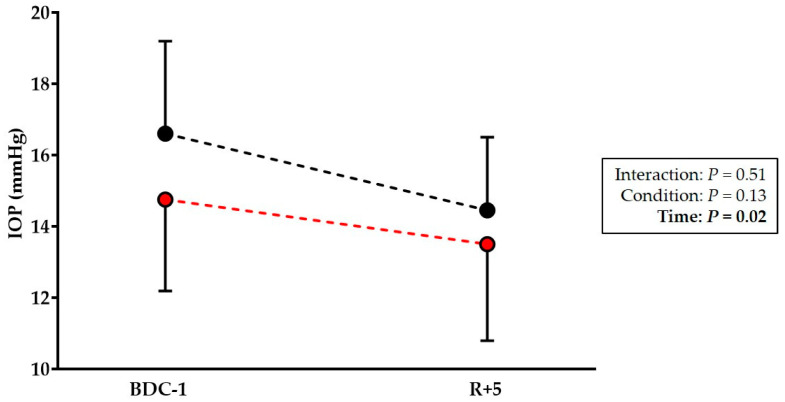
Intraocular pressure (IOP) measurements before (BDC-1) and after (R+5) head-down bed rest in control (black circle) and antioxidant cocktail (red circle) groups. Data are expressed as mean ± SD.

Two-way ANOVA indicated that there was a main effect of time (*p* = 0.02) on the IOP. Post hoc comparisons revealed a non-significant drop in the IOP in the CON group at R+5 (*p* = 0.07). It must be emphasized that eight of the ten volunteers had a diminished IOP. However, in the NUT group, the IOP remained unchanged at R+5 (*p* = 0.37) (Figure 4).

## 4. Discussion

Our study shows that 60 days of HDBR induces ophthalmological changes, such as a decrease in CRT and an enlarged ONSD. It should be pointed out that two volunteers presented peripapillary edema but recovered 1 and 2 months later. Finally, the antioxidant cocktail failed to dampen these ophthalmological modifications.

Degraded distant and near visual acuity has been described in some astronauts. Indeed, astronauts return to Earth with several ocular changes: ONSD enlargement, hyperopic shift, choroidal folds, posterior globe flattening, etc. [1,2]. Furthermore, there is a case report of an astronaut who underwent repeated long-duration spaceflights and developed some recurrent ophthalmological changes [9]. Persistent optic disc edema has been described in a case study of an astronaut over 6 months post-flight with high total retinal thickness (TRT) values, measured by OCT, which persisted well beyond one year post-flight [10]. OCT scans of 15 astronauts after long-duration spaceflight revealed an increase in the global circumpapillary RNFLT (especially in the inferior quadrant) and TRT (superior, inferior and nasal quadrants) [11]. It also appeared that global TRT changes persisted 1 year after spaceflight in 11 astronauts who spent a mean duration of ~170 days on board the International Space Station [12]. The same authors depicted that two of the eleven astronauts developed a greater global TRT with optic disc edema and one of them presented choroidal folds that worsened over the 1-year mission [13]. In our study, the CRT tended to be reduced after HDBR (eight of the nine subjects had a reduced CRT after HDBR). Although a reduced CRT is correlated with better visual acuity [14], these subtle changes are unlikely to impact visual acuity. Moreover, the RNFLT did not change, which confirmed our previous findings from a 21-day HDBR [8]. However, in contrast to this previous study, two subjects presented peripapillary edema. We observed, in these two subjects, a sharp increase in the RNFLT in the temporal superior (7.0 and 12.0 µm), nasal (4.5 and 11.5 µm) and nasal superior (5.5 and 16.5 µm) quadrants. In terrestrial analogues, such as HDBR, similar findings were observed. Indeed in a case report of a volunteer, the peripapillary retinal thickness, determined by spectralis OCT, was greater (~5%) after a 30-day HDBR with no visible clinical signs of optic disc edema [15]. Nevertheless, in a 30-day strict HDBR (i.e., without a standard pillow), optic disc edema was described with a rise in the peripapillary TRT in all quadrants (inferior, superior, nasal and temporal). The rise in the TRT was even greater than those observed either after a 14- or 70-day HDBR [16,17]. The time duration of the experiment has an impact on the amount of optic disc swelling since the authors observed a greater peripapillary retinal thickness after a 70-day HDBR compared to a 14-day HDBR [16]. The authors also revealed greater differences in the peripapillary TRT after a 30-day strict HDBR in 11 healthy volunteers compared to those assessed during short-duration spaceflight (~37 days) in 20 astronauts [18]. Another 6° strict HDBR performed in 24 healthy subjects showed an increase in TRT at HDT58 (~9%) and remained higher during recovery (~7%) [19]. These ocular changes could be due to an HDBR-induced cranial fluid shift.

Mader et al. [1] described that in most astronauts who underwent 6 months of spaceflight, there was a globe flattening determined by MRI. Lumbar punctures, performed in astronauts with disc edema, showed an opening pressure up to ~20 mmHg and persisted over weeks after spaceflight. Moreover, after long-term spaceflight, the astronauts with globe flattening exhibited the highest ONSD values (7.2 mm) compared to those with no globe flattening (5.8 mm). Similarly, astronauts with nerve kinking had the highest ONSD values (7.5 mm) compared to those with no nerve kinking (5.9 mm) [20]. Consecutive long-term missions to the International Space Station may also have an impact on the ONSD dilatation, as documented by MRI in an astronaut [9]. In-flight ONSD values from a preliminary study, obtained with 2D ultrasound in 13 astronauts, showed a rise of ~11% compared to pre-flight values, with recovery post-flight [21]. A case report also described, in an astronaut who underwent a 6-month spaceflight to the International Space Station, an increase in the ONSD that persisted after the space mission [10]. However, in similar conditions, quantitative MRI showed a preserved ONSD in 10 astronauts, indicating that the intracranial pressure (ICP) did not reach a pathological level. It is worth noting that one subject with optic disc edema also presented a distended ONSD; however, this elevation was small compared to patients with an increased ICP [22]. In the horizontal position, the normal ICP in healthy subjects lies between 7 and 15 mmHg and an ICP greater than 20 mmHg defines intracranial hypertension [23]. It has become widely accepted that the ONSD indirectly reflects the ICP [24,25,26]. However, no cut-off value of ONSD exists and many studies proposed a threshold to predict intracranial hypertension [27,28,29]. However, in our study, the highest ONSD values (HDBR+57; CON group: 5.2 ± 0.4 mm, NUT group: 5.0 ± 0.7 mm) do not seem to reach the threshold observed in intracranial hypertension or high ICP values. The onset of ONSD enlargement may be multifactorial. While it is possible that this distension might be a localized episodic event, the retrobulbar space of the ONS may distend under the influence of hydrostatic transmittance of cerebrospinal fluid pressure within the subarachnoid space [30,31]. After long-duration spaceflight, some crewmembers presented stagnant or reverse flow in the internal jugular vein [32]; however, the occlusion of the venous drainage pathways may also lead to a rise in the ICP [33]. Thus, an altered venous and lymphatic drainage may disrupt the absorption and sequestration of the cerebrospinal fluid leading to a distended subarachnoid space [27,30]. Moreover, it would appear that subjects with a deficiency in circulating concentrations of folate and B12 might experience the development of ocular symptoms [34].

The IOP seems to be affected immediately after exposure to microgravity. Indeed, an 8-day German Spacelab mission showed a rise in the IOP of ~20–25% after only a few minutes in microgravity [35]. During the 10-day Spacelab D2 mission, the IOP increased dramatically and doubled during the 1st day, with a return to the baseline value on the 4th day [36]. Similar findings of IOP data from six shuttle missions were observed in 11 astronauts [30]. In our study, we observed a non-significant decrease (~13%) in the IOP after HDBR (eight of ten subjects had a reduced IOP). However, in our previous study, the IOP was preserved after a 21-day HDBR [8]. The use of different tonometers (Perkins vs. Goldmann) and the different days of measurements (R+1 vs. R+5) are likely to explain these differences. Nevertheless, the IOP was reduced in 25 female volunteers after a 7-day HDBR. The authors assumed that hypovolemia, induced by a thoraco-cephalic fluid shift during HDBR, provoked ocular dehydration or systemic cardiovascular and hormonal changes, leading to a reduced IOP [37]. Another assumption is that persistent compensatory mechanisms intervene during the supine position, such as reduced aqueous humour production, leading to a decreased IOP [38]. A previous report noted a similar trend with a reduced post-flight IOP [39]. A case report of a healthy male subject confirmed this tendency and showed a reduction in the IOP by ~27% after a 30-day HDBR [15]. Although Taibbi et al. [16] described a rise in the IOP during 14- and 70-day HDBR (+1.42 and +1.79 mmHg, respectively), the IOP was normalized after the experiment. Previously, one of the hypotheses proposed was that the microgravity-induced fluid shift led to a rise in the ICP that was greater than the rise in the IOP, leading to a decreased translaminar pressure gradient [30,40,41,42].

Antioxidant intake is commonly used as a therapy against glaucoma [43] with the help of different protective mechanisms such as preventing ganglion cell loss or reducing the IOP [44]. In this study, the antioxidant cocktail neither improved nor exacerbated the reduction in CRT or the ONSD enlargement induced by HDBR. Oxidative stress contributes to endothelial dysfunction and may lead to ocular outcomes in astronauts such as optic disc edema, choroidal folds, globe flattening, ONS thickening, etc. [45]. As previously argued by Zwart et al. [45], the intake of antioxidant precursors may not be sufficient to dampen ophthalmic syndrome. In fact, Tousoulis et al. [46] did not observe any effect of vitamins and antioxidants on homocysteine-induced endothelial dysfunction in hypertensive patients. Thus, an exogenous supply would be insufficient to increase intracellular reservoirs of antioxidants [45] and consequently provoke an impact on HDBR-induced ocular modifications. Creating a placebo that sufficiently resembles the intake of an antioxidant cocktail proved very challenging. Hence, the lack of a placebo in this study may impact our findings. However, the similar conditions of this study have most likely compensated for this limitation. Moreover, the small number of subjects may dampen the significance of our findings, especially about the ONSD. In this way, our study may lack statistical power to detect significant differences between groups. These results could have become significant with more subjects included. Only males participated in this study; thus, we obviously cannot extrapolate our findings to females. Indeed, the effect of hormonal changes, such as estrogen, may influence ophthalmological data. In future studies, a deeper understanding of gender effects on ophthalmological adaptations to microgravity will be essential to minimize the health risks. Furthermore, additional ophthalmological parameters should also be studied, such as retinal thickness, to identify possible macular edema and the ganglion cell complex, providing information about potential glaucoma.

In all, HDBR provoked ophthalmological changes such as a reduced CRT and an enlarged ONSD. Importantly, two subjects presented peripapillary edema but recovered 1 and 2 months later. Finally, the antioxidant cocktail failed to prevent the HDBR-induced neuro-ophthalmological modifications.

## Data Availability

All datasets generated for this study are included in the manuscript.

## References

[1] Mader T.H., Gibson C.R., Pass A.F., Kramer L.A., Lee A.G., Fogarty J., Tarver W.J., Dervay J.P., Hamilton D.R., Sargsyan A. (2011). Optic disc edema, globe flattening, choroidal folds, and hyperopic shifts observed in astronauts after long-duration space flight. Ophthalmology.

[2] Lee A.G., Mader T.H., Gibson C.R., Tarver W., Rabiei P., Riascos R.F., Galdamez L.A., Brunstetter T. (2020). Spaceflight Associated Neuro-Ocular Syndrome (SANS) and the Neuro-Ophthalmologic Effects of Microgravity: A Review and an Update. NPJ Microgravity.

[3] Saveko A., Bekreneva M., Ponomarev I., Zelenskaya I., Riabova A., Shigueva T., Kitov V., Abu Sheli N., Nosikova I., Rukavishnikov I. (2023). Impact of different ground-based microgravity models on human sensorimotor system. Front. Physiol..

[4] Ong J., Lee A.G., Moss H.E. (2021). Head-Down Tilt Bed Rest Studies as a Terrestrial Analog for Spaceflight Associated Neuro-Ocular Syndrome. Front. Neurol..

[5] Pavy-Le Traon A., Heer M., Narici M.V., Rittweger J., Vernikos J. (2007). From Space to Earth: Advances in Human Physiology from 20 Years of Bed Rest Studies (1986–2006). Eur. J. Appl. Physiol..

[6] Kennedy A.R. (2004). Biological Effects of Space Radiation and Development of Effective Countermeasures. Life Sci. Space Res..

[7] Convertino V.A. (2002). Planning strategies for development of effective exercise and nutrition countermeasures for long-duration space flight. Nutrition.

[8] Kermorgant M., Hammoud S., Mahieu L., Geeraerts T., Beck A., Bareille M.-P., Soler V., Pavy-Le Traon A., Quintyn J.C. (2021). Effects of Resistance Exercise with or without Whey Protein Supplementation on Ocular Changes after a 21-Day Head-Down Bed Rest. Life.

[9] Mader T.H., Gibson C.R., Pass A.F., Lee A.G., Killer H.E., Hansen H.C., Dervay J.P., Barratt M.R., Tarver W.J., Sargsyan A.E. (2013). Optic disc edema in an astronaut after repeat long-duration space flight. J. Neuro-Ophthalmol..

[10] Mader T.H., Gibson C.R., Otto C.A., Sargsyan A.E., Miller N.R., Subramanian P.S., Hart S.F., Lipsky W., Patel N.B., Lee A.G. (2017). Persistent Asymmetric Optic Disc Swelling After Long-Duration Space Flight: Implications for Pathogenesis. J. Neuro-Ophthalmol..

[11] Patel N., Pass A., Mason S., Gibson C.R., Otto C. (2018). Optical Coherence Tomography Analysis of the Optic Nerve Head and Surrounding Structures in Long-Duration International Space Station Astronauts. JAMA Ophthalmol..

[12] Macias B.R., Patel N.B., Gibson C.R., Samuels B.C., Laurie S.S., Otto C., Ferguson C.R., Lee S.M.C., Ploutz-Snyder R., Kramer L.A. (2020). Association of Long-Duration Spaceflight with Anterior and Posterior Ocular Structure Changes in Astronauts and Their Recovery. JAMA Ophthalmol..

[13] Macias B.R., Ferguson C.R., Patel N., Gibson C., Samuels B.C., Laurie S.S., Lee S.M.C., Ploutz-Snyder R., Kramer L., Mader T.H. (2021). Changes in the Optic Nerve Head and Choroid Over 1 Year of Spaceflight. JAMA Ophthalmol..

[14] Santos A.R., Gomes S.C., Figueira J., Nunes S., Lobo C.L., Cunha-Vaz J.G. (2014). Degree of decrease in central retinal thickness predicts visual acuity response to intravitreal ranibizumab in diabetic macular edema. Ophthalmologica.

[15] Taibbi G., Kaplowitz K., Cromwell R.L., Godley B.F., Zanello S.B., Vizzeri G. (2013). Effects of 30-Day Head-Down Bed Rest on Ocular Structures and Visual Function in a Healthy Subject. Aviat. Space Environ. Med..

[16] Taibbi G., Cromwell R.L., Zanello S.B., Yarbough P.O., Ploutz-Snyder R.J., Godley B.F., Vizzeri G. (2016). Ocular Outcomes Comparison Between 14- and 70-Day Head-Down-Tilt Bed Rest. Investig. Ophthalmol. Vis. Sci..

[17] Laurie S.S., Macias B.R., Dunn J.T., Young M., Stern C., Lee S.M.C., Stenger M.B. (2019). Optic Disc Edema after 30 Days of Strict Head-down Tilt Bed Rest. Ophthalmology.

[18] Laurie S.S., Lee S.M.C., Macias B.R., Patel N., Stern C., Young M., Stenger M.B. (2020). Optic Disc Edema and Choroidal Engorgement in Astronauts During Spaceflight and Individuals Exposed to Bed Rest. JAMA Ophthalmol..

[19] Laurie S.S., Greenwald S.H., Marshall-Goebel K., Pardon L.P., Gupta A., Lee S.M.C., Stern C., Sangi-Haghpeykar H., Macias B.R., Bershad E.M. (2021). Optic disc edema and chorioretinal folds develop during strict 6° head-down tilt bed rest with or without artificial gravity. Physiol. Rep..

[20] Kramer L.A., Sargsyan A.E., Hasan K.M., Polk J.D., Hamilton D.R. (2012). Orbital and intracranial effects of microgravity: Findings at 3-T MR imaging. Radiology.

[21] Sirek A.S., Garcia K., Foy M., Ebert D., Sargsyan A., Wu J.H., Dulchavsky S.A. (2014). Doppler ultrasound of the central retinal artery in microgravity. Aviat. Space Environ. Med..

[22] Rohr J.J., Sater S., Sass A.M., Marshall-Goebel K., Ploutz-Snyder R.J., Ethier C.R., Stenger M.B., Martin B.A., Macias B.R. (2020). Quantitative magnetic resonance image assessment of the optic nerve and surrounding sheath after spaceflight. NPJ Microgravity.

[23] Czosnyka M., Pickard J.D. (2004). Monitoring and interpretation of intracranial pressure. J. Neurol. Neurosurg. Psychiatry.

[24] Geeraerts T., Dubost C. (2009). Theme: Neurology—Optic nerve sheath diameter measurement as a risk marker for significant intracranial hypertension. Biomark. Med..

[25] Dubost C., Le Gouez A., Zetlaoui P.J., Benhamou D., Mercier F.J., Geeraerts T. (2011). Increase in optic nerve sheath diameter induced by epidural blood patch: A preliminary report. Br. J. Anaesth..

[26] Liu D., Li Z., Zhang X., Zhao L., Jia J., Sun F., Wang Y., Ma D., Wei W. (2017). Assessment of intracranial pressure with ultrasonographic retrobulbar optic nerve sheath diameter measurement. BMC Neurol..

[27] Geeraerts T., Newcombe V.F., Coles J.P., Abate M.G., Perkes I.E., Hutchinson P.J., Outtrim J.G., Chatfield D.A., Menon D.K. (2008). Use of T2-weighted magnetic resonance imaging of the optic nerve sheath to detect raised intracranial pressure. Crit. Care.

[28] Soldatos T., Karakitsos D., Chatzimichail K., Papathanasiou M., Gouliamos A., Karabinis A. (2008). Optic nerve sonography in the diagnostic evaluation of adult brain injury. Crit. Care.

[29] Montorfano L., Yu Q., Bordes S.J., Sivanushanthan S., Rosenthal R.J., Montorfano M. (2021). Mean value of B-mode optic nerve sheath diameter as an indicator of increased intracranial pressure: A systematic review and meta-analysis. Ultrasound J..

[30] Stenger M.B., Tarver W.J., Brunstetter T., Gibson C.R., Laurie S.S., Lee S.M.C. (2017). Evidence Report: Risk of Spaceflight Associated Neuro-Ocular Syndrome (SANS).

[31] Fall D.A., Lee A.G., Bershad E.M., Kramer L.A., Mader T.H., Clark J.B., Hirzallah M.I. (2022). Optic nerve sheath diameter and spaceflight: Defining shortcomings and future directions. NPJ Microgravity.

[32] Marshall-Goebel K., Laurie S.S., Alferova I.V., Arbeille P., Auñón-Chancellor S.M., Ebert D.J., Lee S.M.C., Macias B.R., Martin D.S., Pattarini J.M. (2019). Assessment of Jugular Venous Blood Flow Stasis and Thrombosis During Spaceflight. JAMA Netw. Open.

[33] Beggs C.B. (2013). Venous hemodynamics in neurological disorders: An analytical review with hydrodynamic analysis. BMC Med..

[34] Zwart S.R., Gibson C.R., Mader T.H., Ericson K., Ploutz-Snyder R., Heer M., Smith S.M. (2012). Vision changes after spaceflight are related to alterations in folate- and vitamin B-12-dependent one-carbon metabolism. J. Nutr..

[35] Draeger J., Schwartz R., Groenhoff S., Stern C. (1993). Self-Tonometry under Microgravity Conditions. Clin. Investig..

[36] Draeger J., Schwartz R., Groenhoff S., Stern C. (1994). Self tonometry during the German 1993 Spacelab D2 mission. Ophthalmologe.

[37] Chiquet C., Custaud M.-A., Pavy-Le Traon A., Millet C., Gharib C., Denis P. (2003). Changes in Intraocular Pressure during Prolonged (7-Day) Head-Down Tilt Bedrest. J. Glaucoma.

[38] Mader T.H., Taylor G.R., Hunter N., Caputo M., Meehan R.T. (1990). Intraocular pressure, retinal vascular, and visual acuity changes during 48 hours of 10 degrees head-down tilt. Aviat. Space Environ. Med..

[39] Gibson C.R., Mader T., Caputo M., Taylor G., Hunter N., Meehan R. (1990). Effects of microgravity and 2G on simultaneous intraocular pressure and retinal vessel caliber changes. Aviat. Space Environ. Med..

[40] Eklund A., Jóhannesson G., Johansson E., Holmlund P., Qvarlander S., Ambarki K., Wåhlin A., Koskinen L.O., Malm J. (2016). The pressure difference between eye and brain changes with posture. Ann. Neurol..

[41] Tymko M.M., Boulet L.M., Donnelly J. (2017). Intracranial pressure in outer space: Preparing for the mission to Mars. J. Physiol..

[42] Jóhannesson G., Eklund A., Lindén C. (2018). Intracranial and Intraocular Pressure at the Lamina Cribrosa: Gradient Effects. Curr. Neurol. Neurosci. Rep..

[43] Garcia-Medina J.J., Rubio-Velazquez E., Lopez-Bernal M.D., Cobo-Martinez A., Zanon-Moreno V., Pinazo-Duran M.D., Del-Rio-Vellosillo M. (2020). Glaucoma and Antioxidants: Review and Update. Antioxidants.

[44] Jabbehdari S., Chen J.L., Vajaranant T.S. (2021). Effect of dietary modification and antioxidant supplementation on intraocular pressure and open-angle glaucoma. Eur. J. Ophthalmol..

[45] Zwart S.R., Gibson C.R., Gregory J.F., Mader T.H., Stover P.J., Zeisel S.H., Smith S.M. (2017). Astronaut ophthalmic syndrome. FASEB J..

[46] Tousoulis D., Bouras G., Antoniades C., Marinou K., Papageorgiou N., Miliou A., Hatzis G., Stefanadi E., Tsioufis C., Stefanadis C. (2011). Methionine-induced homocysteinemia impairs endothelial function in hypertensives: The role of asymmetrical dimethylarginine and antioxidant vitamins. Am. J. Hypertens..

